# Applying fuzzy qualitative comparative analysis to identify typical symptoms of COVID-19 infection in a primary care unit, Rio de Janeiro, Brazil

**DOI:** 10.1038/s41598-022-26283-y

**Published:** 2022-12-24

**Authors:** Nádia Cristina Pinheiro Rodrigues, Mônica Kramer de Noronha Andrade, Joaquim Teixeira Netto, Denise Leite Maia Monteiro, Valéria Teresa Saraiva Lino, Eric Gustavo Ramos Almeida

**Affiliations:** 1 Escola Nacional de Saúde Pública Sérgio Arouca/Fundação Oswaldo Cruz, Rio de Janeiro, Rua Leopoldo Bulhões, 1480, 21041210 Brazil; 2 Instituto de Medicina Social Hesio Cordeiro/Universidade do Estado do Rio de Janeiro, Rua São Francisco Xavier, 524-7th floor, Block D, Rio de Janeiro, CEP: 21041-210 Brazil; 3 Faculdade de Ciências Médicas, Universidade do Estado do Rio de Janeiro, Av. Prof. Manuel de Abreu, 444-2º Andar, Rio de Janeiro, RJ CEP: 20550-170 Brazil

**Keywords:** Health services, Public health

## Abstract

This study aims to identify a set of symptoms that could be predictive of SARS-CoV-2 cases in the triage of Primary Care services with the contribution of Qualitative Comparative Analysis (QCA) using Fuzzy Sets (fsQCA). A cross-sectional study was carried out in a Primary Health Care Unit/FIOCRUZ from 09/17/2020 to 05/05/2021. The study population was suspect cases that performed diagnostic tests for COVID-19. We collected information about the symptoms to identify which configurations are associated with positive and negative cases. For analysis, we used fsQCA to explain the outcomes “being a positive case” and “not being a positive case”. The solution term “loss of taste or smell and no headache” showed the highest degree of association with the positive result (consistency = 0.81). The solution term “absence of loss of taste or smell combined with the absence of fever” showed the highest degree of association (consistency = 0,79) and is the one that proportionally best explains the negative result. Our results may be useful to the presumptive clinical diagnosis of COVID-19 in scenarios where access to diagnostic tests is not available. We used an innovative method used in complex problems in Public Health, the fsQCA.

## Introduction

In the period 2020–2021, the impact of the Pandemic COVID-19 in Brazil was catastrophic: 21,975,989 cases and 611,481 deaths^1^, leading to high healthcare costs and increasing social inequality. In Brazilian large cities, where the context of inequities is more visible, there were high rates of morbidity and mortality from COVID-19^2^. One of the challenges has been the access to diagnostic tests for SARS-Cov2 and consequently, many cases are notified based on the clinical symptoms. At the beginning of the pandemic period, the main symptoms of the disease reported are fever, cough, fatigue, dyspnea, sore throat, headache, chest pain, myalgia, conjunctivitis, diarrhea, and anosmia^3,4^. As new variants of the COVID-19 virus have emerged since the beginning of the pandemic, symptoms have changed over time. To date, five variants of the virus have been identified in Brazil and across the world since 2021: alpha, beta, gamma, delta, and omicron^5^.

For the Wuhan strain, the original version of the virus, which appeared at the beginning of the pandemic in March 2020, the symptoms of Covid-19 considered the most common are fever, cough, fatigue, and loss of taste or smell. Less common are headache, sore throat, red or itchy eyes, diarrhea, skin rash, or discoloration of fingers or toes. The most serious symptoms include difficulty breathing or shortness of breath, loss of speech or mobility, mental confusion, and chest pain. In most cases, only a few of these symptoms appear, but the vast majority remain asymptomatic. In the symptom profile of the alpha variant (which emerged in September 2020 in the UK), loss or alteration of smell, loss or alteration of taste, fever, cough, chills, loss of appetite, and muscle pain are the main symptoms found. For the beta variant (which appeared in October 2020 in South Africa), the presence of fever, cough, sore throat, respiratory distress, diarrhea, vomiting, body pain, and fatigue stands out. In the symptom profile of the delta variant (which emerged in October 2020 in India), the prominent symptoms are a runny nose, headache, sneezing, sore throat, persistent cough, and fever. For the gamma variant (detected in November 2020 in travelers from Brazil), the most frequent symptoms are fever, cough, sore throat, shortness of breath, diarrhea, vomiting, body pain, tiredness, and fatigue. The symptom profile of the Omicron variant (which emerged in November 2021 in South Africa) is mainly marked by extreme tiredness, body aches, headache, and sore throat^5^.

Currently, in the post-pandemic scenario, some questions remain, such as how much vaccination protects against infections and serious diseases, what would be the consequences of the increase in the transmissibility of the disease, and the immune evasion of some strains^6,7^. In addition, other specific symptoms, such as cardiac dysfunction in patients with COVID-19, also worry health professionals and are also targets of investigation^8,9^.

Brazil is a huge country with different regions but has a public health system, the Unique Health System (SUS), in which the Primary Health Care Service is responsible for the first access to population health assistance. In this scenario, this study aims to identify a set of symptoms that could be predictive of SARS-CoV-2 cases in patient triage of Primary Care Service with the contribution of the Qualitative Comparative Analysis (QCA) using Fuzzy Sets (fsQCA).

## Data and methods

In this section, we discuss the design setting and source of data and then discuss the methods of data collection and methods used for the analyses. This study was conducted in a Primary Care Unit/FIOCRUZ in Manguinhos, a high degree of social vulnerability neighborhood of Rio de Janeiro City/Brazil. Manguinhos has a population of about 40,000 inhabitants distributed within an area of 261.84 hectares. Manguinhos is a territory of great human mobility, including migrations. This scenario facilitates the importation of cases from other locations and other Brazilian states, which contributes to the increase in the transmission of COVID-19^10^.

### Data

From 09/17/2020 to 05/05/2021, of the 3277 suspected cases reported by the local epidemiological surveillance service, 1807 cases were tested for COVID-19 by molecular test RT-PCR and/or Rapid Antigen Test. Symptoms were collected based on the definition of suspected cases of Sars-Cov-2 by the Brazilian Ministry of Health^11^ to explore which symptom configurations were associated with positive and negative COVID-19 cases. The symptoms collected in this study were self-reported by the suspected cases.

The response variables were “being a positive case”, and “not being a positive case”. The explanatory variables were the following symptoms: sore throat, fever, cough, loss of taste, loss of smell, headache, runny nose, arthralgia, myalgia, diarrhea, abdominal pain, vomiting, fatigue, and respiratory distress. Using variables “loss of taste” and “loss of smell”, we build a new variable “loss of taste or smell”. Symptom selection was guided by theoretical criteria.

### Methods

This is a cross-sectional study whose data analysis used the fuzzy approach of the QCA technique. The QCA technique has great potential to elucidate complex associative relationships^12^. The method seeks to identify the configurations of factors related to the outcome and eliminate irrelevant factors, through a systematic technique of correspondences and contrasts between cases^13–16^.

Although all the study variables are dichotomous, we choose to use fsQCA instead of Crisp-Set (csQCA), whose study variables need to be dichotomized, due to a large number of cases, and since fsQCA can do a more refined analysis. The study variables were calibrated according to fsQCA using degrees of membership that ranged from 0 (fully out membership) to 1 (fully in membership), with 0.5 representing a crossover point (neither in nor out)^13,17^.

From the information of the response variable “being a positive case”, we build a new response variable “not being a positive case”, applying the negation operation. In this way, two fuzzy models were built using the Quine-McCluskey algorithm, one for the response variable, “being a positive case” and another for the response variable “not being a positive case”.

**Figure 1:**
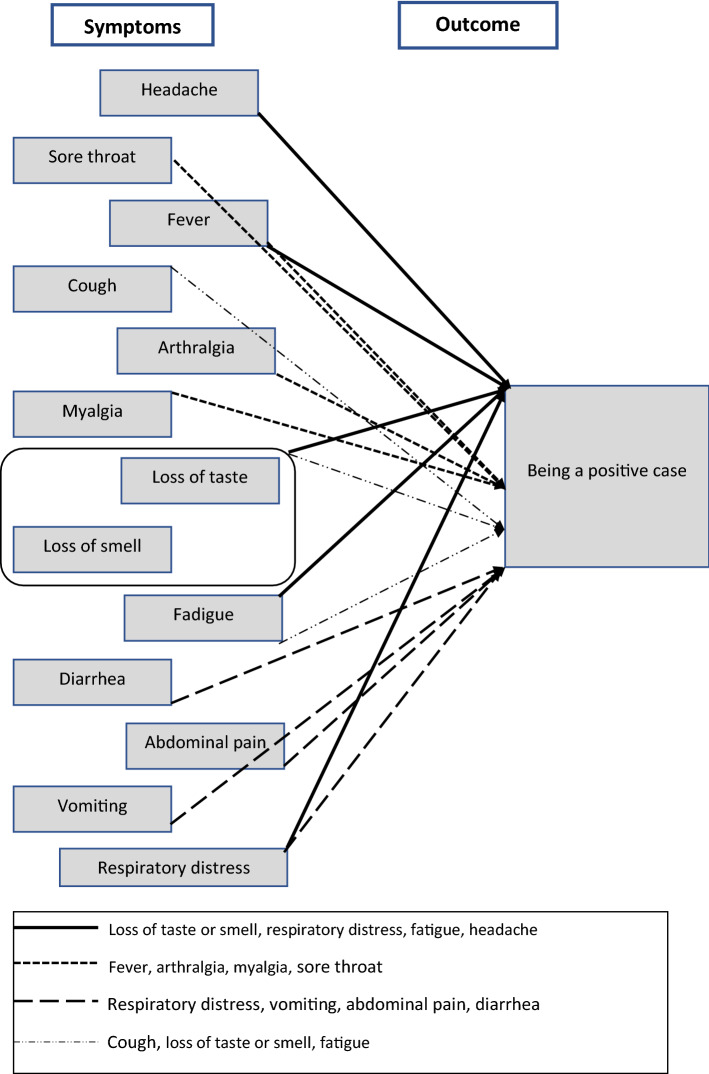
Study hypothesis about the set of symptoms associated with COVID-19.

Besides the symptoms sore throat, fever, cough, loss of taste, loss of smell, headache, runny nose, arthralgia, myalgia, diarrhea, abdominal pain, vomiting, fatigue, and respiratory distress, we built a new variable “loss of taste or smell”, applying the logical operator OR, which represents the union of the set of cases with loss of taste and loss of smell. The abundance of symptoms to consider led us to test, based on the literature, different sets of symptoms that could be associated with the two outcomes (Fig. 1). We tried to keep a low number of factors to avoid obtaining only an individualized description for each case, instead of a parsimonious analysis^13^. The factors included in both models were: fever, headache, loss of taste or smell, fatigue, and respiratory distress. For all these five symptoms it was also applied the negation operation. It was used as a cut-off point to compose the models: minimum consistency of 0.80 and minimum frequency of 10 cases.

To present the results, the parsimonious solution of the models was used. The crude gross and unique coverage and the consistency of each combination of factors were calculated, in addition to the coverage and consistency of the solution of each model. Consistency measures the degree to which the solution terms and the solution as a whole are subsets of the investigated outcome, while coverage measures how much of the investigated outcome is covered (or explained) by each solution term and the solution as a whole^18^.

The fscqa software version 3.0 was used in the analyses.

The study was approved by the Ethics Committee of FIOCRUZ, CAAE: 43691721700005240. The authors declare that all methods were performed following the relevant guidelines of the Research Ethics Committee.

### Ethical statement

The authors declare that the publication entitled “Applying Fuzzy Qualitative Comparative Analysis to identify typical symptoms of COVID-19 infection in a Primary Care Unit, Rio de Janeiro, Brazil”, resulting from research involving data from the surveillance service of the Centro de Saúde Escola Germano Sinval Faria, is in agreement with Brazilian legislation (Resolution 510, of April 7, 2016, of the National Health Council of Brazil (CNS, 2019), and was approved by the Ethics Committee of FIOCRUZ, CAAE: 43691721700005240, with the approval of the waiver of the Free and Informed Consent of research, given the justification of the difficulty of access to patients registered in the surveillance service. According to Art. 14 of Section I of Resolution 510: “Art. 14. When it is impossible to carry out the Free and Informed Consent process, the waiver of this process must be justifiably requested by the responsible researcher to the CEP/CONEP System for consideration.”

## Results

Table 1 presents all possible combinations of symptoms that were reported by at least 10 of the participants. Of the 32 possible configurations, 20 and 24 met the criterion of consistency and minimum frequency of cases, for the outcomes “being a positive case” and “not being a positive case”, respectively (Table 1).

**Table 1:** Truth table with presence and absence of outcome “positive case”.

Respiratory distress		Loss of taste OR smell	Fever	Headache	Fatigue	Number of cases > 9 membership	Consistency as a subset of the outcome
**Outcome**							
**Positive case**							
0		1	1	1	0	145	0.8
0		1	1	1	1	105	0.82
0		1	0	1	1	92	0.86
0		1	0	0	0	90	0.88
0		1	1	0	0	49	0.95
1		0	0	0	0	47	0.84
1		0	0	0	1	44	0.88
0		1	0	0	1	39	0.93
1		1	1	1	1	39	0.91
1		0	0	1	0	38	0.86
1		0	0	1	1	37	0.86
1		0	1	1	1	37	0.89
1		0	1	0	1	34	0.95
1		0	1	1	0	26	0.91
1		1	1	1	0	22	0.93
1		1	0	1	1	22	0.94
0		1	1	0	1	21	0.96
1		1	1	0	1	16	0.98
1		0	1	0	0	15	0.96
1		1	0	0	1	10	0.99
**NOT positive**							
0		0	0	1	0	594	0.84
0		0	0	0	0	486	0.82
0		0	0	1	1	226	0.88
0		0	1	1	1	161	0.82
0		0	0	0	1	150	0.87
0		1	1	1	1	105	0.82
0		1	0	1	1	92	0.81
0		0	1	0	1	89	0.88
0		1	1	0	0	49	0.84
1		0	0	0	0	47	0.96
1		0	0	0	1	44	0.93
1		1	1	1	1	39	0.92
0		1	0	0	1	39	0.89
1		0	0	1	0	38	0.97
1		0	0	1	1	37	0.98
1		0	1	1	1	37	0.94
1		0	1	0	1	34	0.9
1		0	1	1	0	26	0.96
1		1	1	1	0	22	0.96
1		1	0	1	1	22	0.96
0		1	1	0	1	21	0.94
1		1	1	0	1	16	0.94
1		0	1	0	0	15	0.97
1		1	0	0	1	10	0.96

Consistency > 0.8 and number of cases > 9 membership.

Table 2 presents all the solutions to the analysis of the two fuzzy models. These solutions indicate the typical configurations associated with each outcome. The outcome “being a positive case” shows that four symptom configuration terms were sufficient to classify the suspected cases as positive: respiratory distress; loss of taste or smell and fatigue; loss of taste or smell and fever; and, loss of taste or smell and absence of headache. However, the solution term “loss of taste or smell and no headache” showed the highest degree of association with the outcome “being a positive case” (consistency = 0.81). For the outcome “not being a positive case”, the results also indicated four configuration terms: respiratory distress, fatigue, no loss of taste or smell, and no fever; no headache and fever but a loss of taste or smell. It is observed that the solution term “absence of loss of taste or smell combined with the absence of fever” showed the highest degree of association (consistency = 0,79) and is the one that proportionally best explains the result “not be positive” (gross coverage = 0.61 and single coverage = 0.36) (Table 2).

**Table 2:** Symptom configurations associated with positive and negative COVID-19 cases.

Configurations	Coverage		Consistency
	Raw	Unique	
**For the outcome: “being a positive case”**			
Respiratory distress	0.25	0.07	0.57
Loss of taste or smell and absence of headache	0.25	0.04	0.81
Loss of taste or smell and fever	0.30	0.06	0.70
Loss of taste or smell and fadigue	0.28	0.04	0.71
Solution coverage	0.48		
Solution consistency	0.58		
**For the outcome: “not being a positive case”**			
Respiratory distress	0.19	0.02	0.74
Fatigue	0.37	0.11	0.67
No loss of taste or smell and no fever	0.61	0.36	0.79
No headache and fever, but a loss of taste or smell	0.09	0.00	0.76
Solution coverage	0.79		
Solution consistency	0.71		

## Discussion

### Main finding of this study

Our results indicate that loss of taste or smell was present in three of the four combinations of symptoms associated with positive cases of COVID-19. Although most settings related to positive cases of COVID-19 contain the symptom "loss of taste or smell", in cases that did not test positive for COVID-19, this symptom also appears in a smaller portion, but in this case, it does not is associated with fever. Respiratory distress was present both in positive and negative cases, although to a lesser extent in negative cases. The isolated symptom of fatigue was not able to configure a positive case of Sars-Cov-2, but when this presented in combination with the loss of smell or taste, it configured a combination of symptoms associated with the presence of the infection.

### What is already known on this topic

There is a wide range of symptoms reported by people who have had COVID-19. Anyone can have mild or severe symptoms of the disease, even if they do not belong to any specific risk group. The period of onset of symptoms is between two to 14 days after exposure to the virus. Fever, chills, cough, difficulty breathing, fatigue, body aches, headache, loss of taste or smell, sore throat, nasal congestion, nausea, diarrhea, appetite loss, and chills are some of these reported symptoms^19,20^.

The sudden and isolated loss of smell or taste, in the absence of inflammatory diseases of the upper airways, such as allergic rhinitis, chronic rhinosinusitis, nasal polyposis, and other conditions, seems to be a warning sign to health professionals of the suspicion of COVID-19^21^. Our results showed that the loss of taste or smell in the absence of the headache would be a configuration of symptoms associated with the presence of COVID-19. Headache, unlike fever, has been reported less frequently in scientific articles describing the manifestations of COVID-19^22^. On the other hand, headache is among the most common symptoms reported in inflammatory disorders of the upper respiratory tract and is often confused with migraines^23^.

An important health surveillance strategy to reduce disease transmission is its rapid detection as early as possible so that cases can be isolated and treated^24^. The investigation of a possible case of COVID-19 is usually based on a combination of clinical parameters that the patient presents (e.g., fever, shortness of breath, and loss of smell and taste). It is from the observed clinical parameters that the search and infection control policies are triggered^25^. Given the scarcity of resources in many countries, testing, in most cases, is only performed if the patient's clinical parameters lead to suspicion of the disease.

Considering the scarce information about studies to identify a set of symptoms for diagnosing COVID-19 cases in the Primary Care Service, this study intends to contribute to the clinical diagnosis of COVID-19 in this scenario. In our results, loss of taste or smell was present in three of the four combinations of symptoms associated with positive cases of COVID-19, which were similarly reported in several studies^21,26^, however, most of them were carried out in different settings like hospitals, outpatients clinics or symptoms tracker apps^27,28^ and a small part, in Primary Care^29^. Altered taste perception (dysgeusia) in Sars-Cov-2 patients is widely reported in studies and can be found in up to 44% of cases^30^.

Both positive and negative cases of COVID-19 contain the symptom "loss of taste or smell", although, in the last, it appears in a smaller portion and is not associated with fever. What could explain the "loss of taste or smell" appearing in both positive and negative patients for COVID-19 would be the duration of the symptom, which usually remains for a few weeks after the patient becomes negative^31^.

Respiratory distress was present both in positive and negative cases, although to a lesser extent in negative cases. The isolated symptom of fatigue was not able to configure a positive case of Sars-Cov-2, but when this presented in combination with the loss of smell or taste, it configured a combination of symptoms associated with the presence of the infection. Fatigue is a common symptom in those with symptomatic COVID-19 and also persists after COVID-19^32^. Perhaps the fact that both respiratory distress and fatigue are associated not only with Sars-Cov-2 but also with other diseases, like, cardiopulmonary, renal, gastrointestinal, oncology, and infectious diseases^33–36^, may explain why these symptoms are not associated solely with positive cases of Sars-Cov-2.

In this study, Qualitative Comparative Analysis Method was chosen due to its capacity to analyze the combinations of signs and symptoms to evaluate the clinical presentation of COVID-19 and model these relationships to obtain subsets that explain a given outcome. It is a usual method applied for social and political research but more recently, it is utilized to respond to some questions related to public health^37,38^.

### What this study adds

Our results may be useful to guide the diagnosis of Sars-Cov-2 in scenarios where testing is not possible, contributing to increasing knowledge about which sets of symptoms could predict the disease. More comprehensive studies, involving different locations, using other analysis techniques could contribute to improving access to the diagnosis of COVID-19.

### Limitations of this study

As a limitation, it was not possible to obtain reliable information on the duration of symptoms in the cases at the time of performing the diagnostic test, so part of the tests may have been performed outside the indicated period for testing, which may have compromised the result. Another issue that was not considered was the type of virus variant, given that the different variants have specific peculiarities, the symptoms may have varied during the collection period. However, despite these limitations, these results can contribute to diagnostic guidance in locations that do not have access to diagnostic tests.

The symptoms used in this study were self-reported by the participants, so they present a certain degree of subjectivity. Loss of taste or smell, for example, despite its subjectivity, was reported in a National Survey in the United States by about 38% of suspected COVID-19 patients and was included by the CDC as one of the main symptoms associated with the disease^39^.

## Supplementary Information

Supplementary Information.

## Data Availability

All data generated or analyzed during this study are included in this published article.
